# Analysis of the *Trichuris suis* excretory/secretory proteins as a function of life cycle stage and their immunomodulatory properties

**DOI:** 10.1038/s41598-018-34174-4

**Published:** 2018-10-29

**Authors:** Louis-Philippe Leroux, Mohamad Nasr, Rajesh Valanparambil, Mifong Tam, Bruce A. Rosa, Elizabeth Siciliani, Dolores E. Hill, Dante S. Zarlenga, Maritza Jaramillo, Joel V. Weinstock, Timothy G. Geary, Mary M. Stevenson, Joseph F. Urban, Makedonka Mitreva, Armando Jardim

**Affiliations:** 1 Institute of Parasitology McGill University, Sainte-Anne-de-Bellevue, QC Canada; 2 Centre for Host-Parasite Interaction (CHPI), Montreal, Canada; 3 Institut National de la Recherche Scientifique (INRS)-Institut Armand-Frappier (IAF), Laval, QC Canada; 4 Division of Experimental Medicine, Department of Medicine, McGill University, Montreal, QC Canada; 5 Department of Microbiology and Immunology, McGill University, Montreal, QC Canada; 6 McDonnell Genome Institute, Washington University in, St. Louis, MO USA; 7 United States Department of Agriculture, Beltsville, MD USA; 8 Division of Gastroenterology-Hepatology, Department of Internal Medicine, Tufts Medical Center, Boston, MA USA; 9 Division of Infectious Diseases, Department of Internal Medicine, Washington University School of Medicine, St. Louis, MO USA

## Abstract

Parasitic worms have a remarkable ability to modulate host immune responses through several mechanisms including excreted/secreted proteins (ESP), yet the exact nature of these proteins and their targets often remains elusive. Here, we performed mass spectrometry analyses of ESP (TsESP) from larval and adult stages of the pig whipworm *Trichuris suis* (Ts) and identified ~350 proteins. Transcriptomic analyses revealed large subsets of differentially expressed genes in the various life cycle stages of the parasite. Exposure of bone marrow-derived macrophages and dendritic cells to TsESP markedly diminished secretion of the pro-inflammatory cytokines TNFα and IL-12p70. Conversely, TsESP exposure strongly induced release of the anti-inflammatory cytokine IL-10, and also induced high levels of nitric oxide (NO) and upregulated arginase activity in macrophages. Interestingly, TsESP failed to directly induce CD4^+^ CD25^+^ FoxP3^+^ regulatory T cells (T_reg_ cells), while OVA-pulsed TsESP-treated dendritic cells suppressed antigen-specific OT-II CD4^+^ T cell proliferation. Fractionation of TsESP identified a subset of proteins that promoted anti-inflammatory functions, an activity that was recapitulated using recombinant *T. suis* triosephosphate isomerase (TPI) and nucleoside diphosphate kinase (NDK). Our study helps illuminate the intricate balance that is characteristic of parasite-host interactions at the immunological interface, and further establishes the principle that specific parasite-derived proteins can modulate immune cell functions.

## Introduction

The incidence of immune-mediated disorders in industrialized or “westernized” countries has increased dramatically over the past century^1–3^. For instance, cases of Crohn’s disease and ulcerative colitis, collectively called inflammatory bowel disease (IBD), are idiopathic, chronic inflammatory disorders of the gastrointestinal tract^4^. The incidence of IBD has increased several fold over the past few decades^2,5^. This general trend in immune-related disorders is concomitant with a significant decrease in the incidence of infectious diseases due to antibiotic use, vaccination, improved hygiene, and overall better socioeconomic conditions^1–3^. It is believed that environmental factors have contributed to the increased incidence of these diseases. Alterations in our pattern of exposure to microorganisms and helminths could play a role as well. This concept, dubbed the “hygiene hypothesis”^3^, is supported by growing epidemiological evidence showing that helminths, multicellular parasitic worms colloquially referred to as “old friends”^6^, play a protective role by modulating the capacity of the host to mount an aberrantly strong immune response to normal immune challenges, as well as, in part, by altering the gut microbial flora^2,7–9^.

Helminths or molecules derived from these organisms are being explored as therapeutic agents to treat immune-related diseases. Clinical trials using the hookworm *Necator americanus* have shown encouraging results for the treatment of celiac disease^10,11^. The porcine whipworm *Trichuris suis* has also gained attention as a potential therapeutic agent^7,12^.

*T. suis* is a soil-transmitted swine parasite^13^. Ova released in the feces undergo embryogenesis and develop into first stage larvae (L_1_). Upon ingestion by a host, the larvae go through four molts (L_2_, L_3_, and L_4_) and develop into the adult stage (L_5_) over a period of 40–45 day in the gastrointestinal tract^14^. Although *T. suis* is closely related to the human whipworm *T. trichiura*, ingestion of *T. suis* ova in most cases leads only to a non-fertile self-limiting colonization in humans^15^.

Helminth infections polarize host immunity towards a Th2 response, which is required for worm expulsion^16^, with a concomitant downregulation of Th1-mediated responses, Th17 cells, an increased production of IL-10 and TGF-β by regulatory T (T_reg_) cells, and the induction of regulatory dendritic cells and alternatively-activated macrophage (AAMΦ) (reviewed in^17^). A number of studies have reported the characterization of excretory/secretory (ES) products with immunomodulatory functions from various parasitic worms, including *Trichinella spiralis*^18–21^, *Heligmosomoides polygyrus*^22–25^, *Teladorsagia circumcincta*^26^, *Mesocestoides corti*^27^, and *N. americanus*^28^. Several studies have also reported similar activity in *T. suis* ES proteins (TsESP)^29,30^ and soluble worm extracts^31–35^. Immunomodulation of host immunity has been recently reported using recombinant *Ancylostoma caninum* tissue inhibitor metalloprotease (AIP-2); this protein promotes expansion of T_reg_ cells, which suppress experimental asthma^36^. In addition, recombinant serine protease inhibitor (serpin) from *Trichinella pseudospiralis* alters macrophage polarization^37^. These studies demonstrate that specific molecules released by helminth parasites can shape host innate and adaptive immune responses. However, the molecular mechanisms driving these events are not clear.

Here, we carried out genome-wide transcriptomic analyses of *T. suis* larval stages and adult worms to identify differentially expressed genes. Proteomic analysis also was performed to profile ESP proteins released by the different life stages of *T. suis*. The immunomodulatory properties of TsESP were examined using murine bone marrow-derived dendritic cells (BMDC) and macrophages (BMDM). Chromatographic separation of TsESP permitted the identification of immunomodulatory proteins, and recombinant versions of several TsESP proteins were used to confirm immunomodulatory activity.

## Results

### *T. suis* genes and excretory/secretory proteins (TsESP) display stage-specific expression

Genomic sequencing of *T. suis* predicted the presence of 9,832 genes, comparable to *T. muris* and *T. trichura* (9,403 and 9,856, respectively), with an average open reading frame length of 2,384 bp (Supplementary Table S1). To assess changes in gene expression as a function of life cycle stage, transcriptomic analysis was performed on 10-, 16-, 21-, 28-day larvae (L_2_, L_3_, and L_4_, respectively) and 42-day adult worm (L_5_) stages and male or female adult worms and these results were compared to ES products identified by proteomics.

**Figure 1:**
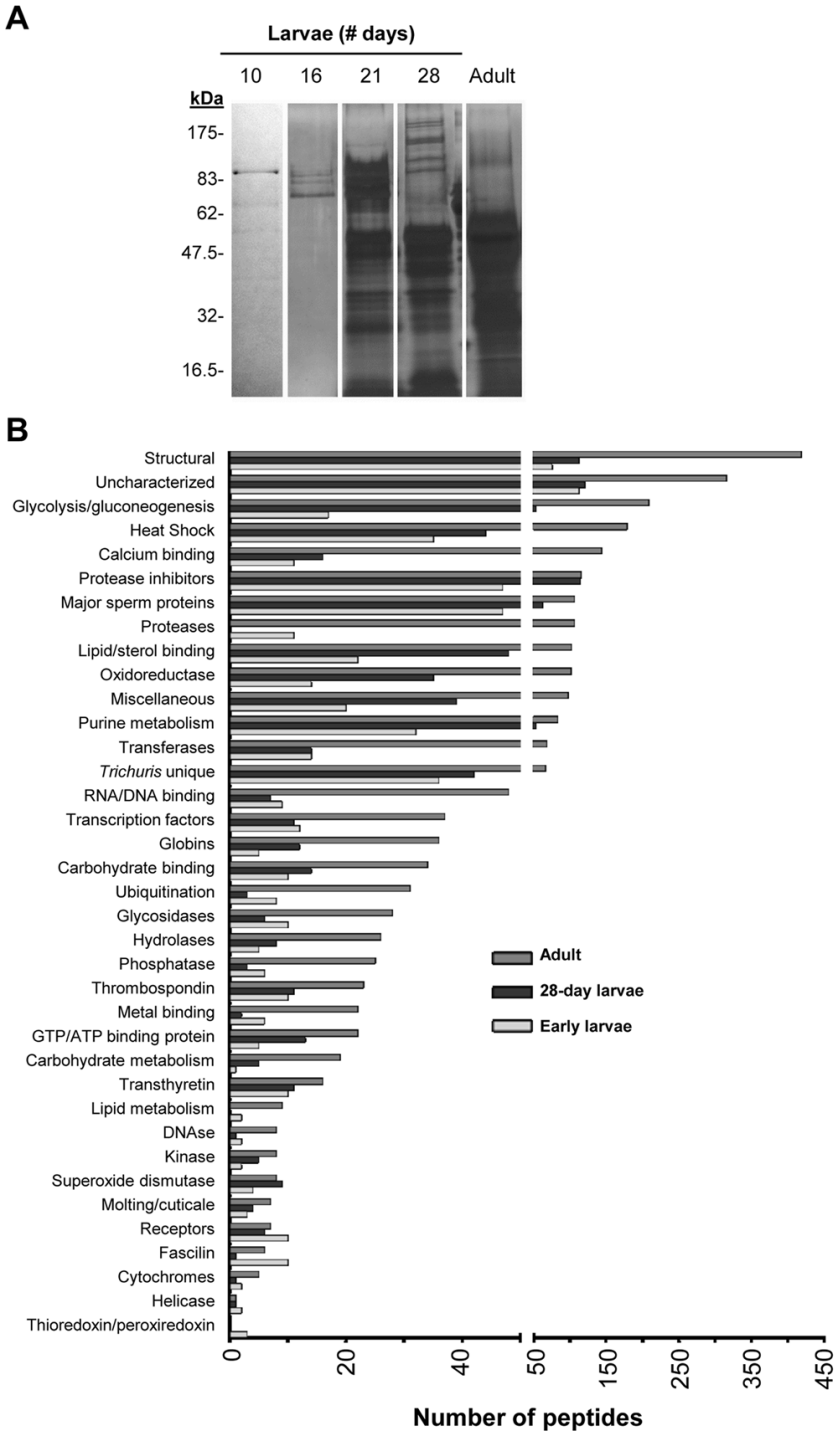
Proteomic analysis of excreted/secreted proteins from various *T. suis* life cycle stages reveal stage-specific expression. (**A**) The diversity of *T. suis* proteins isolated from concentrated serum-free culture medium were analyzed by silver-stained SDS-PAGE prior to subjecting samples to nano LC-MS/MS for protein identification. Full-length scans of SDS-PAGE gels are shown in Supplementary Fig. S3. (**B**) GO term analysis was performed to identify biological function enrichment of proteins identified by LC-MS/MS in early (10-, 16-, 21-day) and 28-day larvae, and adult worms.

To examine ESP released by *T. suis*, parasites isolated from infected pigs at 10, 16, 21, 28, and 42 days post-inoculation and cultivated *in vitro* for up to 72 h. Conditioned medium was collected for protein analysis. SDS-PAGE analysis showed that proteins with MW ranging from ~10–180 kDa were released by 21- and 28-day larvae and adult worms (Fig. 1A). Protein output was notably lower for 10- and 16-day larvae. Proteomic analysis of TsESP isolated from the various life stages collectively identified ~350 proteins using single preparations for 10-, 16-, and 21-day larvae, and three biological replicates for 28-day larvae and adult worms (Supplementary Table S2). Based on Gene Ontology (GO) analysis and biological function, these proteins were classified into 37 groups with structural proteins, glycolytic enzymes, chaperones, proteases, protease inhibitors, and uncharacterized proteins representing the most abundant classes (Fig. 1B). Interestingly, ~20 of the uncharacterized proteins detected in this analysis appeared to be specific to *Trichuris* and shared very little or no sequence homology with other helminth proteins (Supplementary Table S3). Bioinformatic analyses showed that ~26% of the proteins in the TsESP proteomes contained a signal sequence and ~15% contained 1–4 predicted transmembrane domains.

**Figure 2:**
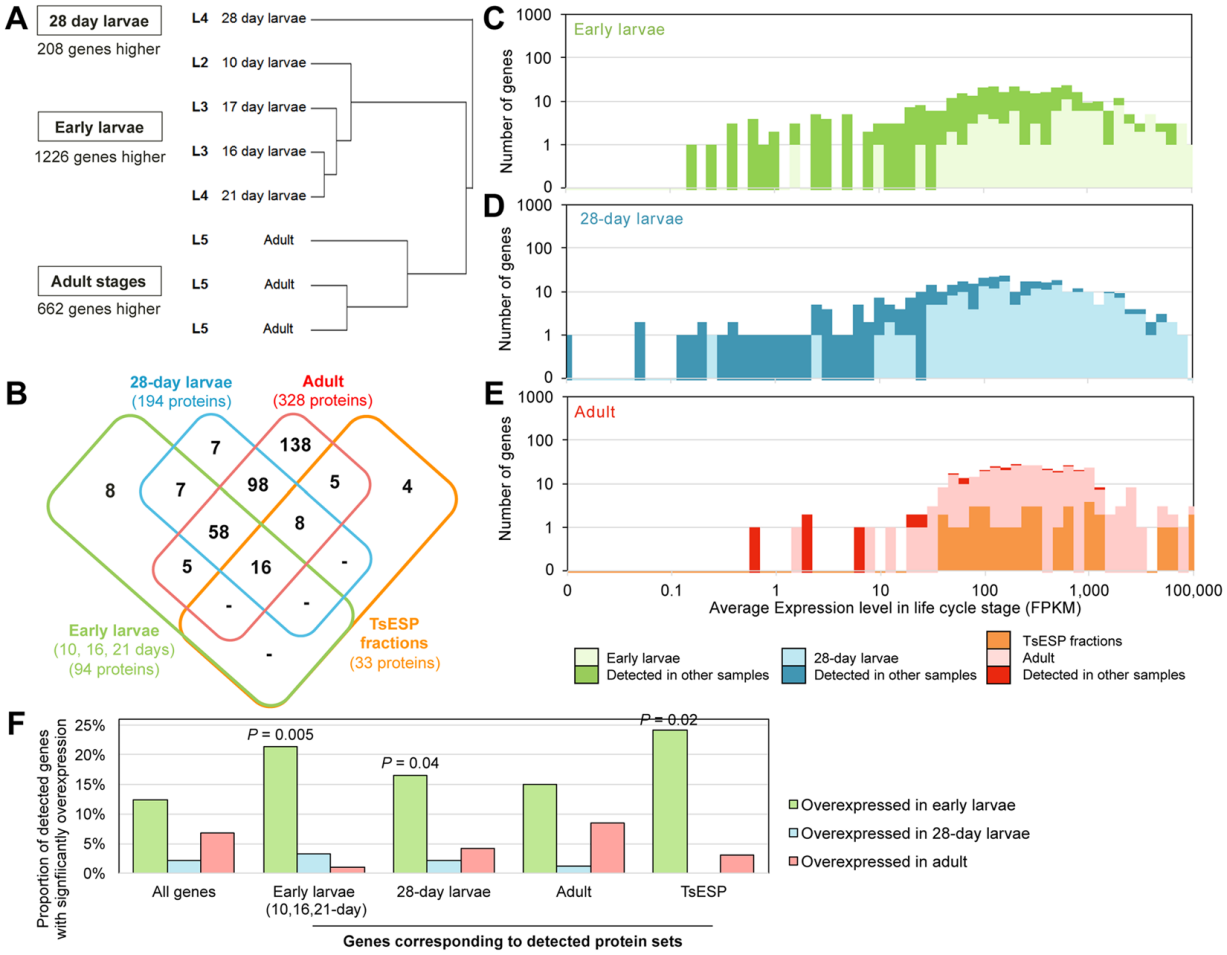
*Trichuris suis* transcriptomic and proteomic analyses. (**A**) Clustering of RNA-Seq samples according to global gene expression patterns (Spearman clustering, average linkage). The number of genes overexpressed in each of 3 sample sets relative to the other sample sets (DESeq2) is indicated. (**B**) Counts of proteins detected across the proteomics datasets. “TsESP fractions” correspond to proteins identified in pooled fractions obtained by gel permeation (see Fig. 6 and Supplementary Table S5). Average gene expression levels of all genes during (**C**) early larvae stages, (**D**) 28-day larvae and (**E**) adult stages, with genes corresponding to detected proteins indicated. (**F**) Differential gene expression among detected protein sets. *P* values represent significant enrichment compared to all genes, according to non-parametric binomial distribution tests.

Differential gene expression analysis was performed using DESeq2 across 3 sample sets consisting of a total of 8 RNA-Seq samples (early larvae: 10-, 16-, 17-, and 21-day; larvae: 28-day; adult, 3 replicates; Supplementary Table S3). This analysis identified, (i) 1,226 genes that were more highly expressed in early larvae and enriched for the GO terms related to ribosome, kinase, and peptidase activity, and less likely than other genes to be *T. suis*-specific (relative to *T. trichiura*, *T. muris*, and 3 host species; 261 genes, *P* = 1 × 10^−7^; Supplementary Table S4), (ii) 208 genes were more highly expressed in 28-day larvae and enriched for GO terms related to cell adhesion and signaling, and motor activity, and (iii) 662 genes more highly expressed in adult worms, enriched for GO terms related to kinase activity, toxin responses, and carbohydrate metabolism, and genes likely to be *T. suis*-specific (226 genes, *P* = 0.0001; Fig. 2A, Supplementary Tables S3, S4). Gene expression levels corresponding to protein sets detected by proteomics (Fig. 2B) are shown for the early larvae (Fig. 2C), the 28-day larvae (Fig. 2D), and the adult (Fig. 2E) RNA-Seq samples, indicating that highly-expressed genes in each stage are more likely to be detected by proteomic analysis; since these proteins appear to be targeted for release into the extracellular environment. Genes corresponding to proteins detected in early larvae TsESP proteomic studies are more likely to be overexpressed in the early larvae in comparison to other life cycle stages (*P* = 0.05), as are those corresponding to proteins detected in the 28-day larvae (*P* = 0.04) and the TsESP (*P* = 0.02; Fig. 2F, Supplementary Table S3). In sum, the proteomic and transcriptomic analyses revealed life stage-specific expression patterns of ES proteins which might reflect the different developmental requirements of the larval stages and suggest that the host immune response is exposed to a concoction of parasite-derived molecules that varies in composition as the parasite develops.

### TsESP inhibit pro-inflammatory cytokine production and induce IL-10 secretion

**Figure 3:**
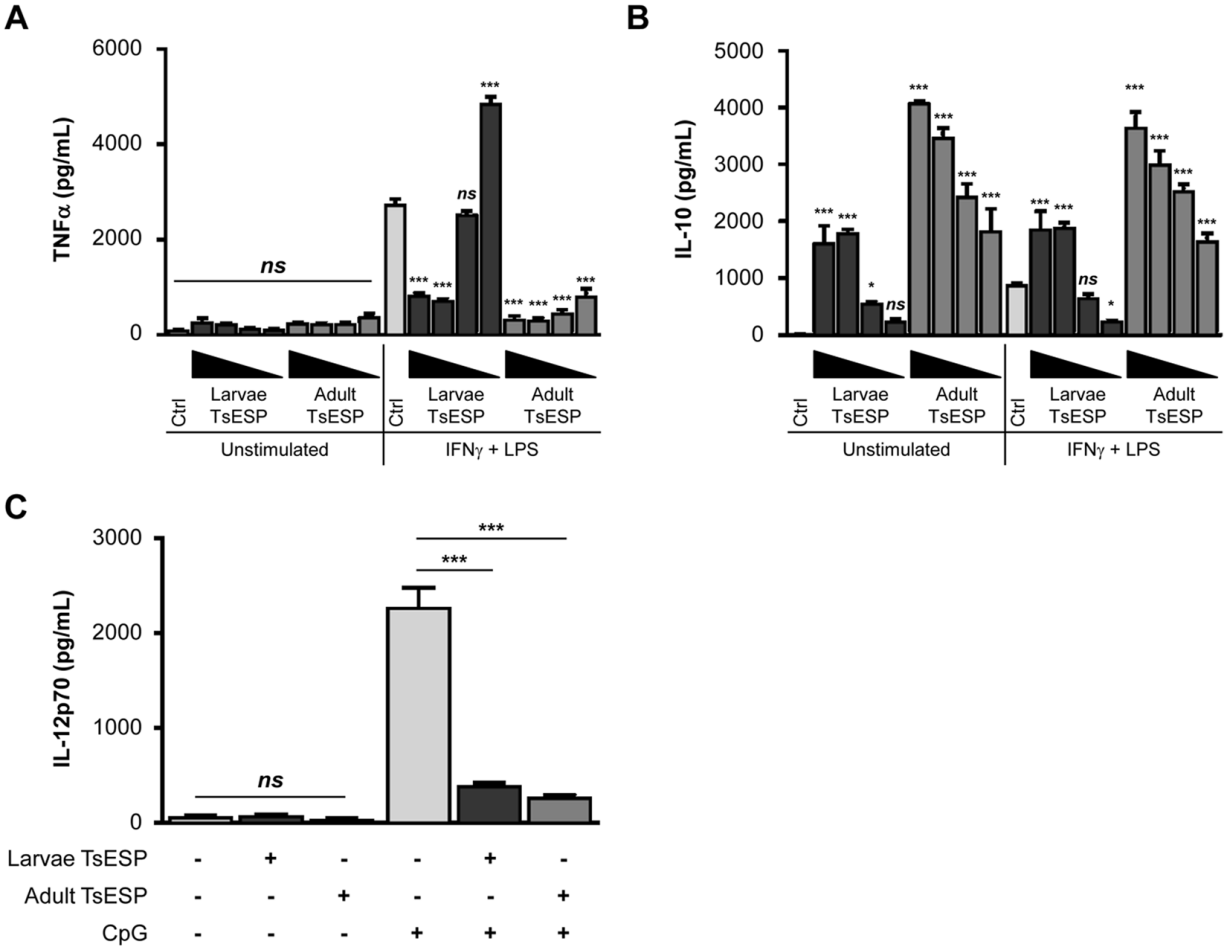
TsESP inhibit pro-inflammatory while inducing anti-inflammatory cytokine release in murine bone marrow-derived immune cells. Bone marrow-derived macrophages (BMDM) and dendritic cells (BMDC) were pretreated with 28-day larvae or adult worm TsESP for 2 h then stimulated with IFNγ and LPS, CpG-ODN, or left unstimulated. (**A**,**B**) TNFα and IL-10 secreted by BMDM, and (**C**) IL-12p70 secreted by BMDC were measured using sandwich ELISA. (**A**,**B**) BMDM were treated with different larvae or adult TsESP concentrations, either 50, 25, 5, or 1 µg/mL, and BMDC cultures were treated with 50 µg/mL TsESP prior to stimulation with CpG-ODN. All samples were prepared in triplicates, and error bars represent standard deviation (SD).

TsESP from adult worms was shown to suppress inflammatory responses in human monocyte-derived dendritic cells^29^. Similarly, TsESP collected from L_1_ hatched *in vitro* reduced clinical signs of allergic airway disease in a murine model^30^. One aim of this study was to directly compare the immunomodulatory effects of TsESP released from larvae and adult worms. To examine these effects, a concentration-dependent experiment was first performed by pre-incubating BMDM with 1–50 µg/mL of TsESP from 28-day larvae and adult worms. The addition of TsESP alone failed to induce TNFα production (Fig. 3A); however, inhibition of TNFα release (>70%; *P* < 0.001) was observed in BMDM cultures pretreated with adult TsESP as low as 1 µg/mL prior to stimulation with interferon-gamma (IFNγ) and LPS, potent inducer of TNFα. Although a similar concentration-dependent response was observed with 28-day larvae TsESP, higher concentrations (≥25 µg/mL) were required to reduce TNFα secretion (>69%; *P* < 0.001) (Fig. 3A), which could reflect differences in the levels of specific immunoactive *T. suis* proteins in these TsESP preparations. In contrast, BMDM pretreated with TsESP alone was sufficient to produce robust levels of the anti-inflammatory cytokine IL-10 (Fig. 3B). Indeed, the addition of ≥25 µg/mL of 28-day larvae or ≥1 µg/mL of adult worm TsESP triggered a marked increase in IL-10 secretion (*P* < 0.001) in unstimulated or IFNγ/LPS-stimulated BMDM cultures. Interestingly, TsESP from adult worms elicited significantly greater IL-10 levels compared to 28-day larvae TsESP when tested at equal concentrations. In addition, pretreatment of BMDC with 28-day larvae and adult worm TsESP reduced IL-12p70 secretion by ~80% (*P* < 0.001) following stimulation of cells with CpG-ODN (Fig. 3C), a TLR9 ligand and known inducer of IL-12p70. Control experiments showed that TsESP treatment alone failed to stimulate production of IL-12p70 by BMDC.

To verify that the immunomodulatory effects were linked to specific protein component(s), TsESP preparations were subjected to extensive proteolysis at an elevated temperature to degrade proteinaceous material. Addition of TsESP to BMDC prior to CpG-ODN stimulation decreased IL-12p70 secretion (Supplementary Fig. S1); however, treatment of 28-day larva or adult worm TsESP with proteinase K^38^ abolished the capacity of TsESP to inhibit IL-12p70 production following CpG-ODN stimulation. In fact, protease treatment dramatically augmented the stimulatory effect on IL-12p70 production, resulting in significantly higher levels of cytokine secretion as compared to CpG-ODN control cultures. Collectively, these data revealed that TsESP from larvae and adult worms altered cytokine profiles in BMDM and BMDC despite concomitant treatment with exogenous pro-inflammatory stimuli. Moreover, these observations not only confirm the immunosuppressive properties of TsESP on innate immune cells, but also highlight the greater potency of adult worm TsESP as compared to day-28 larvae.

### TsESP induce nitric oxide (NO) production and arginase-1 (ARG-1) expression

**Figure 4:**
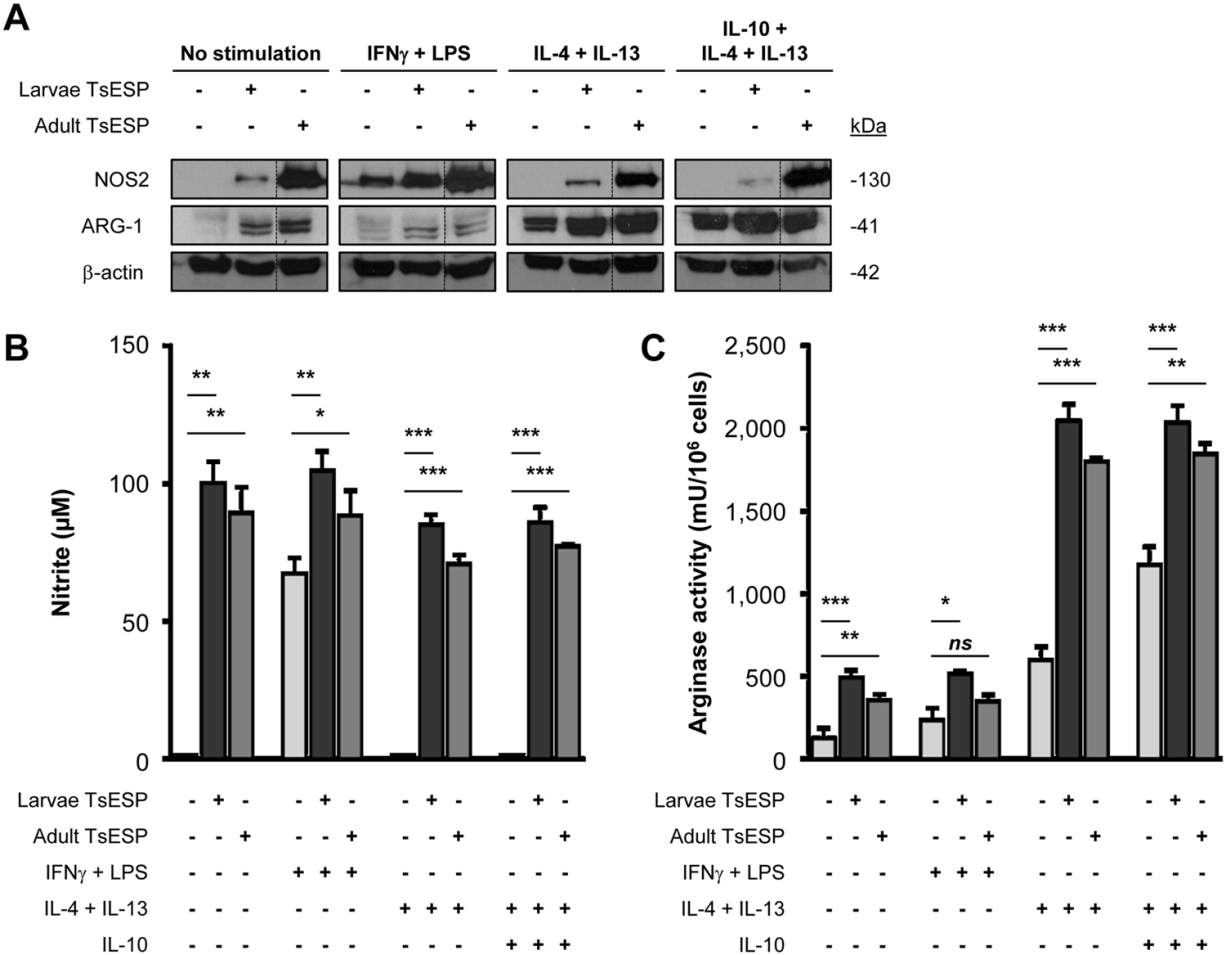
TsESP up-regulate both NOS2 and ARG-1 levels in BMDM. (**A**) Expression of NOS2 (nitric oxide synthase 2) and ARG-1 (arginase 1) in TsESP-treated BMDM cultures was assessed by Western blotting. BMDM were pretreated with 28-day larvae or adult worms TsESP for 2 h, or left untreated. Cultures were stimulated with IFNγ and LPS, a combination of IL-4 and IL-13 with or without IL-10, or left untreated for 18–20 h. Total amounts of β-actin were used as a loading control. Full-length scans of Western blotting films are shown in Supplementary Fig. S3. (**B**) Nitric oxide (NO) levels in the culture supernatant were quantified using the Griess reagent and (**C**) arginase activity was measured in cell lysates. All samples were prepared in triplicate, and error bars represent standard deviation (SD).

Parasitic nematodes skew the polarization of macrophages from classically-activated (M1) to alternatively-activated macrophages or suppressive M2 through different mechanisms^29,39,40^. The general paradigm for macrophage polarization states that M1 produce high levels of NO, while M2 display increased levels of ARG-1^41,42^. Surprisingly, Western blot analyses revealed that treatment of BMDM with 28-day and adult TsESP alone induced expression of nitric oxide synthase 2 (NOS2 or iNOS) and ARG-1 (Fig. 4A). Concomitant treatment of BMDM with a combination of IFNγ and LPS with or without TsESP resulted in robust upregulation of NOS2; however, the levels were notably higher following treatment with adult TsESP. TsESP from 28-day larvae had a less pronounced effect, regardless of the addition of exogenous cytokines or LPS. In contrast, TsESP treatment alone caused an increase in ARG-1 expression, while concomitant addition of IL-4 and IL-13 with or without IL-10 drastically enhanced its expression in TsESP-treated and control cultures.

To determine if increased NOS2 expression resulted in enhanced NO production, levels of NO were estimated in the culture medium by measuring nitrite concentrations. Treatment of BMDM with either 28-day larvae or adult worm TsESP alone was sufficient to triggered a sharp increase in NO levels (*P* < 0.01) (Fig. 4B). Furthermore, NO concentrations were higher in cultures pretreated with TsESP followed by exposure to a combination IFNγ and LPS (*P* < 0.05), factors known to induce NO production^43^. Surprisingly, TsESP-induced NO production by BMDM remained elevated despite treatment with IL-4 and IL-13 with or without IL-10 (*P* < 0.001), both previously shown to inhibit NOS2 transcription^44–46^. Elevated arginase activity was also observed when cells were exposed to TsESP with or without additional IFNγ and LPS treatment (Fig. 4C). Consistent with the increased expression of ARG-1 (Fig. 4A), BMDM treated with IL-4 and IL-13 showed a robust upregulation in arginase activity which was further elevated in the presence of IL-10. Addition of TsESP had a pronounced additive effect with the latter cytokines leading to higher arginase activity as compared to control cultures (*P* < 0.01) (Fig. 4C). The simultaneous upregulation of NOS2 and ARG-1 suggests that TsESP did not polarize BMDM to an M1 or M2 phenotype, as typically defined, but rather towards a phenotype reminiscent of myeloid-derived suppressor cells (MDSC)^47^ with potential suppressive abilities.

### TsESP-polarized myeloid cells suppress antigen-specific CD4^+^ T cell proliferation

**Figure 5:**
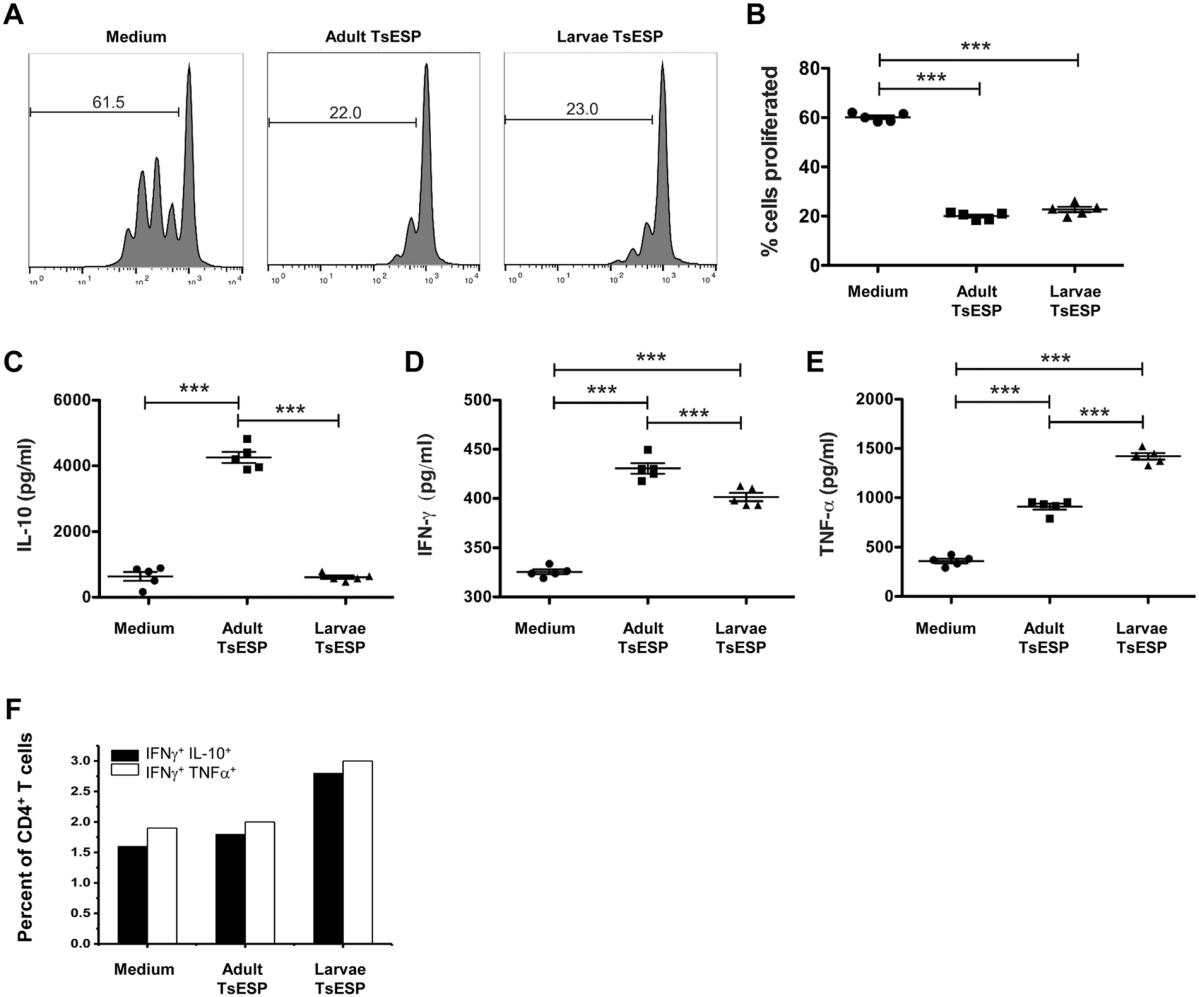
TsESP-polarized bone marrow-derived cells suppress antigen-specific CD4^+^ T cell proliferation. BMDC were pretreated for 2 h with either 28-day larvae or adult TsESP, or left untreated (i.e. medium only), then co-cultured with CD4^+^ T cells in the presence of 1 nM OVA_323–339_ peptide. (**A**) Representative profiles of CFSE dilution of gated CD4^+^ T cells are shown, while proliferation was measured and plotted with five biological replicates for each condition. (**C–E**) Levels of IL-10, IFNγ, and TNFα in supernatants harvested from co-cultures were determined by ELISA. (**F**) CD4^+^ T cells harvested at 48 h from co-cultures were treated with Brefeldin A for the last 4 h of incubation and stained for intracellular IL-10, IFNγ, and TNFα. Histograms represent the staining of TNFα and IL-10 from the CD4^+^IFNγ^+^ cell population. CD11c^+^ BMDC had negligible staining for ICS for any of the cytokines (data not shown).

Given the phenotype observed with TsESP-treated myeloid cells, we sought to determine if these cells could alter antigen-specific expansion of CD4^+^ T cells. Co-cultures of BMDC pulsed with OVA_323–339_ peptide and OT-II CD4^+^ T cells induced robust T cell division as shown by the dilution of the CFSE signal (Fig. 5A), which corresponded to a ~60% increase in T cell proliferation (Fig. 5B). However, treatment of BMDC with adult worm or larval TsESP prior to co-culturing caused a marked and significant inhibition of OT-II CD4^+^ T cell division (~40% reduction in cell proliferation as compared to medium control) after a 72 h incubation; Fig. 5A,B). Determination of cytokine levels in the supernatants revealed high levels of IL-10 in cultures containing BMDC pretreated with adult worm TsESP, while supernatants from the medium control and BMDC pretreated with 28-day larvae TsESP contained only minimal levels of IL-10 (Fig. 5C). A similar analysis showed that cultures containing BMDC pretreated with TsESP either from adult worms or day-28 larvae had increased levels of IFNγ and TNFα (Fig. 5D,E).

To assess which cells in BMDC:OT-II CD4^+^ T cell co-cultures released cytokines (IL-10, IFNγ, TNFα), intracellular cytokine staining was performed after a 48 h incubation. Cells were stained for CD11c or CD4 cell surface markers to differentiate BMDC and T cells, respectively, and doubly stained to detect intracellular levels of IFNγ and IL-10 or IFNγ and TNFα. Dual intracellular staining of T cell populations following stimulation with OVA peptide presented by BMDC showed that 2.4–3.5% of CD4^+^ T cells expressed IFNγ (Fig. 5F). Co-staining of the latter population for TNFα confirmed that ~2.0% of the CD4^+^ T cells in the medium control and TsESP-treated cultures expressed IFNγ and TNFα. The frequency of IFNγ^+^ and TNFα^+^ CD4^+^ T cells increased to ~3.0% in cultures treated with TsESP. CD4^+^ T cells expressing both IFNγ and IL-10 were detected at a frequency of ~1.7% in the medium control and 28-day larvae TsESP while the frequency of IFNγ^+^ IL-10^+^ CD4^+^ T cells increased to ~2.8% in the adult worm TsESP-treated cultures, which may account for the notably higher level of IL-10 detected in the culture supernatant following pretreatment of BMDC with adult TsESP (Fig. 5E).

It was previously reported that exposure to ESP from other intestinal nematodes *H. polygyrus* and *T. circumcincta* induced differentiation of naïve splenocytes into CD4^+^ CD25^+^ FoxP3^+^ T_reg_ cells^26^, an effect we did not observe with TsESP treatment (Supplementary Fig. S2). Flow cytometric analyses showed that the expression of the CD25 and FoxP3 markers in concanavalin (Con) A-stimulated splenocytes did not differ when control cultures (4.1 ± 1.4%) were compared to TsESP-treated cultures (28-day larvae 5.3 ± 1.3%; adult worm 5.0 ± 1.5%). In contrast, ESP from adult *H. polygyrus* worms induced a significant increase (14.1 ± 2.7%, *P* < 0.05) in *de novo* CD4^+^ CD25^+^ FoxP3^+^ T cells, as previously reported^26^. Overall, these data suggest that the suppressive effects of TsESP are mediated by TsESP-polarized myeloid cells that limit anti-specific CD4^+^ T cell proliferation without directly inducing T_reg_ cells.

### Fractionation of adult worm TsESP

**Figure 6:**
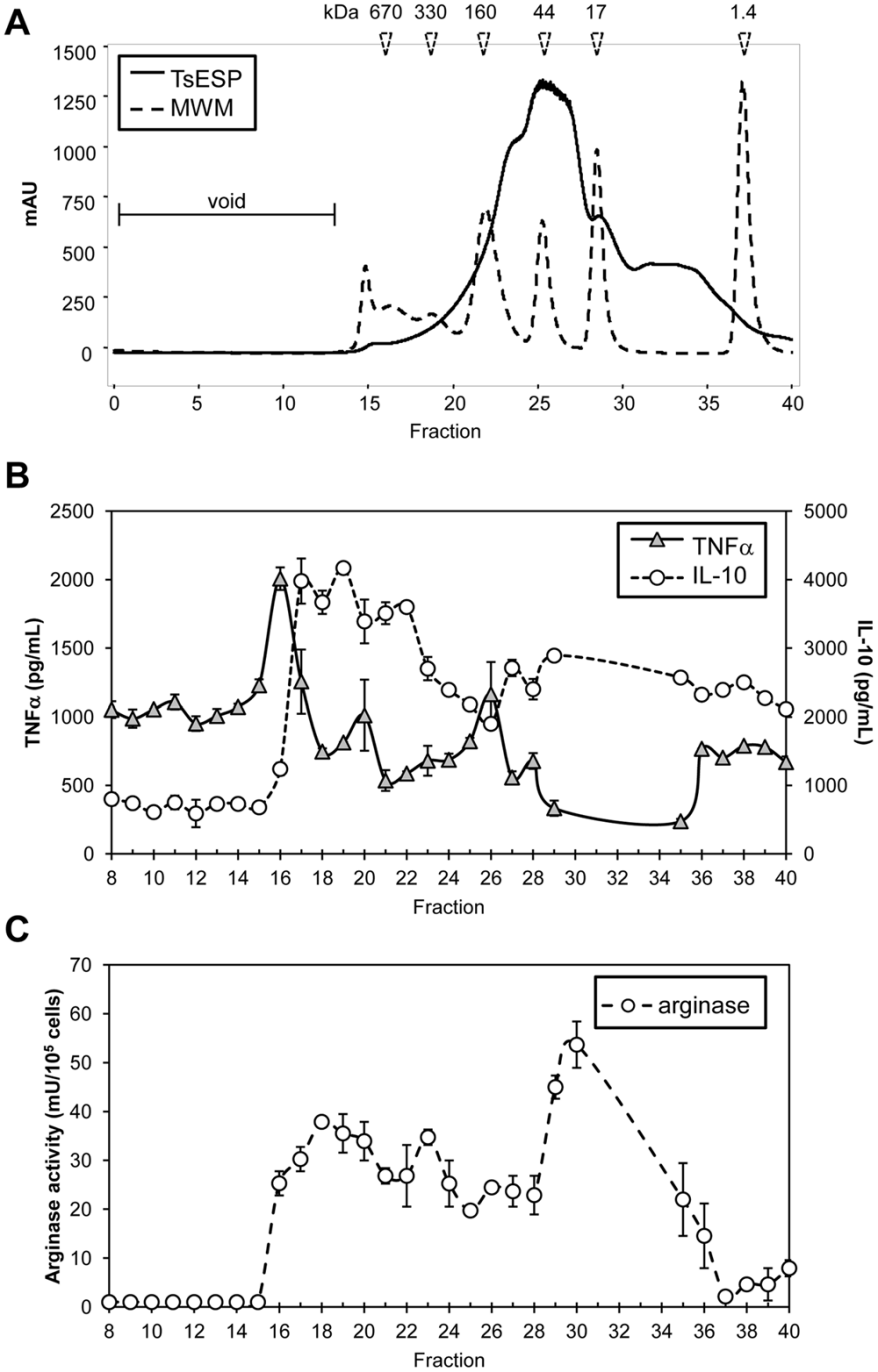
Fractionation of adult TsESP and identification of immunomodulatory protein candidates. (**A**) Approximately 4.0 mg of adult worm TsESP were fractioned by gel permeation chromatography (HPLC). PBS was used as a mobile phase, and 1 mL fractions were collected. Eluting proteins were monitored at 280 nm wavelength. A mixture of proteins of known molecular weights were ran subsequently to allow the estimation of protein sizes. (**B**,**C**) BMDM cultures were treated with ~10 µg of protein collected from each fraction (from Fraction #8 to #40) for 4 h, then stimulated with IFNγ and LPS for 18–20 h. (**B**) Levels of TNFα and IL-10 in the supernatant was quantified by a sandwich ELISA assay, and (**C**) arginase activity was measured in cell lysates.

To identify immunomodulatory proteins in adult TsESP, high-performance liquid chromatography (HPLC) fractionation was performed to separate proteins on the basis of size. Gel permeation chromatography (GPC) revealed that the majority of proteins eluted with apparent MW ranging from ~17–200 kDa (Fig. 6A). To identify bioactive components, aliquots of the GPC fractions were used to treat BMDM cultures followed by IFNγ/LPS stimulation. Levels of IL-10 and TNFα in the supernatant (Fig. 6B) and arginase activity in cells were subsequently quantified (Fig. 6C). TNFα inhibition activity was detected in multiple GPC fractions; however, the most striking decrease was observed in fractions 17–19, 20–21, and 28–31 (Fig. 6B). A similar analysis showed that fractions 16–23 (MW ~ 160–670 kDa) and 28–31 (~10–35 kDa) induced IL-10. Arginase activity was most strongly detected with fractions 18–21 and 28–30 (Fig. 6C), which coincided with IL-10-inducing fractions.

To identify proteins linked to the immunomodulatory activity, the three pools of GPC fractions with highest bioactivity (16–18, 19–21, and 28–30) were subjected to LC-MS/MS analysis. Collectively, 33 proteins were detected across the three GPC pools (Supplementary Table S5). With the exception of ferritin, Sm ribonucleoprotein, aspartyl aminopeptidase, and α-mannosidase 2C1, all other proteins were previously observed in the crude 28-day larvae or adult worm proteomes. In fractions 16–18, angiotensin-converting enzyme was the only protein also detected in the crude 28-day larvae and adult worm proteomes. The bulk of proteins exhibiting TNFα inhibitory and IL-10 stimulating activity were in fractions 19–21; although several of these proteins were also found in fractions 28–30. Among these, there were a number of proteases, glycolytic enzymes, kinases, dehydrogenases, and the iron and cholesterol binding proteins, ferritin and the Niemann-Pick C1 protein, respectively. A number of uncharacterized proteins were also detected, with proteins D918-01198 and D918-10108 being specific to *Trichuris spp*. (Supplementary Table S5).

**Figure 7:**
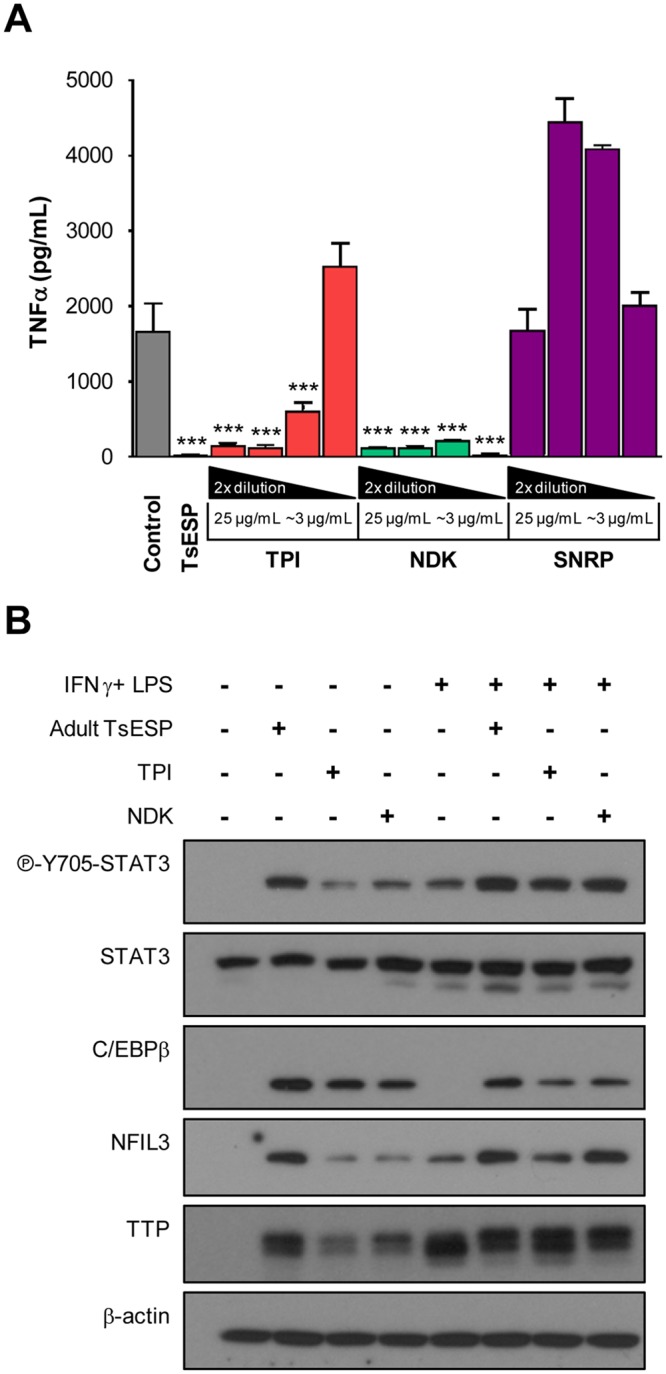
*T. suis* recombinant proteins TPI and NDK proteins inhibit TNFα secretion and elicit anti-inflammatory signaling in BMDM. (**A**) BMDM cultures were treated with different concentrations of recombinant *T. suis* proteins TPI, NDK, or putative protein 505 (two-fold dilutions starting at 25 µg/mL down to ~3 µg/mL) of medium only (i.e. “*Control*”) for 4 h, then stimulated with IFNγ and LPS for 18–20 h. Native, unfractionated adult TsESP were included for comparison. Levels of TNFα were measured by ELISA. Samples were prepared in technical triplicates and results are representative of two independent experiments**. (B**) BMDM cultures were treated with adult worms TsESP (50 µg/mL), recombinant TPI, NDK (both 25 µg/mL), or left untreated for 4 h. Cultures were stimulated with IFNγ and LPS or left untreated for 18–20 h. Phosphorylation of STAT3 (Y705) and expression of C/EBPβ, NFIL-3, and TTP were assessed by Western blotting. Total amounts of β-actin were used as a loading control. Full-length scans of Western blotting films are shown in Supplementary Fig. S3.

Since the amount of protein found in each of the GPC bioactive fractions was limiting, it precluded the possibility of performing additional chromatographic separation steps to obtain single highly purified protein components. Therefore, we generated recombinant versions of several *T. suis* proteins, including triosephosphate isomerase (TPI), nucleoside diphosphate kinase (NDK), and a small nuclear ribonucleoprotein (SNRP), in an *E. coli* expression system to assess and validate their immunomodulatory activity. These three candidates were selected for initial studies since they are found in ESPs from multiple helminths. Bioinformatic analysis showed that *T. suis* SNRP, TPI, and NDK have predicted MWs of 8.0, 27, and 26 kDa and share ~83, 61, and 75% sequence identity with their mammalian homologues, respectively. An interesting feature of the *T. suis* NDK is a 76 amino acid C-terminal extension that is absent from the mammalian homologue, but shares ~40% sequence identity with autophagy related protein 2 from *Trichinella*. Treatment of BMDM with recombinant TPI and NDK inhibited IFNγ/LPS-induced TNFα secretion in a concentration-dependent manner (25 to ~6 µg/mL for TPI, and 25 to ~3 µg/mL for NDK) (Fig. 7A). In contrast, SNRP had no inhibitory activity at the tested concentrations, but instead appeared to potentiate TNFα production.

To examine the cellular mechanisms underlying the anti-inflammatory effects of TPI and NDK, we assessed by Western blots the phosphorylation (activation) and expression of different mediators. Specifically, we monitored the phosphorylation of STAT3 and the expression of CCAAT/enhancer-binding protein β (C/EBPβ), two transcription factors critical in MDSC functions^47^. Treatment of BMDM with adult TsESP, TPI, or NDK without additional stimulation led to the phosphorylation of STAT3 (Y705) and induced expression of C/EBPβ) (Fig. 7B). Also, expression of nuclear factor interleukin-3 regulated (NFIL-3), a target gene of STAT3-mediated transcription and a suppressor of *Il12b* transcription^48^, was induced upon exposure to TsESP. Of note, STAT3, C/EBPβ, and NFIL3 are all important transcription mediators driving the expression of numerous anti-inflammatory genes^48–51^. In addition, a marked increase was observed in the expression of TTP (tristetraprolin), a protein which binds specific mRNAs, such as TNFα and IFNγ^52^, and targets them for degradation. Of note, native TsESP elicited a stronger response for these targets, suggesting that several proteins in this mixture contribute to the effect. In sum, the identification of potential protein candidates supports the concept that specific parasite-derived molecules can be linked to the immunomodulatory properties of TsESP and may promote the immunosuppressive functions of myeloid cells.

## Discussion

Published reports show that parasitic helminths can profoundly modulate immune responses of infected hosts, generally eliciting a Th2-biased response^16^, promoting the production of anti-inflammatory mediators (IL-4, IL-10, IL-25, IL-33, PGE2, TGF-β, TSLP)^24,53–59^, and inducing the expansion of regulatory dendritic cells^39^ and AAMΦ^40^. This skewed immune response is in part due to the immunomodulatory properties of ES products from these parasites^18–26,28–30^. In this study, we have focused on proteins released by *T. suis*; however we recognized that these helminths release other small metabolic products such as PGE2 which are capable of inhibiting pro-inflammatory responses^59^.

Our results with TsESP are largely in agreement with two previous reports, the first of which demonstrated the anti-inflammatory properties of adult TsESP on intestinal epithelial cells (IECs) and monocyte-derived dendritic cells^29^, and the second, which showed the immunosuppressive properties of L_1_ larvae TsESP *in vitro* and in a murine model of allergic airway disease^30^. Our study extends these findings by revealing that TsESP from adult worms is more active than TsESP derived from L_4_ larvae (28-day), which may be linked to stage-specific expression profiles and/or the relative abundance of bioactive proteins, as suggested by the results of our transcriptomic and proteomic analyses. These observations offer insights into changes that occur between the parasite and the host immune system as the larvae develop into adult worms. The subtle yet significant differences in immunomodulatory abilities of different life stages could represent important considerations in the design of a novel therapeutic tool for addressing pathologies associated with immune-mediated disorders.

Since the time that this work was performed, an additional two T. suis genome assemblies (female and male) have been published^60^ (Supplementary Table S1). These two genome assemblies were slightly larger than our assembled genome (71 mbp and 74 mbp for female and male respectively, vs 64 mbp for our assembly) and were annotated with more protein coding genes (14,261 and 14,436, vs 9,832 genes). However, our protein coding gene count was comparable to other published *Trichuris* species (9,403 for *T. muris* and 9,856 for *T. trichiura*^61^), and our genome completion estimation using BUSCO^62^ indicated that our genome was 95% complete similar to the 94.7% completion rate for the other two published versions (Supplementary Table S1), suggesting that we are capturing the complete functional gene set with fewer genes. Furthermore, deduced protein sequence similarity matching using NCBI BLASTp (v2.6.0+) indicated that 90.7% and 90.5% of our genes had matches (E ≤ 10^−5^) in the previously published female and male T. suis genomes, respectively^60^, while the two genomes had 94.5% of genes matching each other. The higher gene count in the published genomes may be the result of gene fragmentation resulting from having more than 10-fold as many contigs in the genome assembly (Supplementary Table S1). Here, we identify biological functions enriched among early larvae (kinases, peptidases, *T. suis*-specific gene functions), 28-day larvae (cell adhesion, motor activity), and adult stage *T. suis* (toxin responses, carbohydrate metabolism, *T. suis*-specific function) using DESeq2 to identify differentially-expressed genes (Supplementary Table S4). The previous genome paper^60^ also had RNA-Seq datasets from L1/L2, L3, L4 and adult stages (in addition to tissue-specific datasets from the male and female posterior body, and the stichosome) but did not perform a similar statistical differential analysis for enrichment for comparison to our results. Hence, our study supports but also complements previous reports by assessing stage-specific gene expression profiles and enrichment of specific biological functions, thus giving greater insight into the developing worms.

Although CpG and LPS (TLR9 and TLR4 ligands, respectively), and IFNγ can trigger distinct signaling events, they also activate common signaling cascades suck as MAPK (e.g. p38, ERK) and increase immune-related gene expression by enhancing the activity of different transcription factors including NFκB and AP-1^63,64^. In our study, we have observed that TsESP treatment was able to inhibit both CpG-mediated IL-12p70 and IFNγ/LPS-mediated TNFα production by innate immune cells. While it remains to be established if the parasite-derived molecules target multiple independent pathways or common downstream effectors of TLR4, TLR9, and IFNγR, our results demonstrate the potency of these molecules to inhibit inflammation without being limited to a single stimulus. This observation is an important consideration especially in the context of treatments of immune-mediated disorders, which are often caused by various molecular cues^4^. In addition, our observations offer valuable clues as to potential signaling events affected which could direct future studies.

Furthermore, our study revealed an interesting effect of TsESP on the polarization of primary macrophages and dendritic cells. Macrophages treated with the parasite products adopted several features akin to myeloid-derived suppressor cells (MDSC), such as the simultaneous expression of NOS2 and ARG1, production of IL-10, higher levels of phosphorylated STAT3, and expression of C/EBPβ (reviewed in^47^). Moreover, TTP, a potent repressor of translation of inflammatory mediators, can be constitutively expressed by tumor-associated macrophages (TAM)^65^ similar to our findings in BMDM treated with TsESP. Another transcription factor induced by TsESP treatment was NFIL3; this protein is important in colonic macrophages to limit inflammation upon exposure to enteric microbiota^48^. Interestingly, *Nfil3*^−/−^ mice develop microbiota-dependent colitis^66^. The expansion of MDSC during infections with *Taenia crassiceps*^67^, *H. polygyrus*^68,69^, and *Schistosoma japonicum*^70^ has been reported; however, evidence for the involvement of ES products in the generation of immunosuppressive myeloid cell subsets in this context would be an important addition to our understanding of host-pathogen interactions. In addition, TsESP-treated dendritic cells display the ability to inhibit antigen-specific CD4^+^ T cell expansion^47^. Curiously, co-culture of TsESP-treated BMDC and OT-II CD4^+^ T cells stimulated with OVA peptide generated IFNγ^+^ IL10^+^ double-positive CD4^+^ T cells. This phenotype has been observed in leishmaniasis^71^ and toxoplasmosis^72^. The underlying mechanism involves an early phase of IFNγ to eradicate the pathogen followed by a regulatory phase or “cytokine switch” during which IL-10 is produced by the same IFNγ^+^ cells, essentially leading to a negative feedback loop to limit inflammation^73^. Hence, TsESP could modulate CD4^+^ T cell responses indirectly by altering the phenotype of antigen presenting cells.

Although there is an unequivocal propensity of TsESP to inhibit inflammatory responses, the identity of the proteins with immune-altering activity has remained largely undetermined for *T. suis* and other nematodes. Using a combination of chromatographic techniques and proteomic analyses, we demonstrated that subsets of TsESP display immunomodulatory capabilities that recapitulate to a large extent effects observed with native TsESP. Three candidates were chosen based on their presence within biologically active GPC fractions and previous literature reports of orthologs in ESP from many other parasitic helminths, namely TPI and NDK, which displayed immunosuppressive activity, and SNRP, which did not have the effects seen with TPI and NDK mimicking the bioactivity observed in native TsESP. TPI is a ubiquitously expressed enzyme that catalyzes the aldose-ketone isomerization of dihydroxyacetone phosphate to glyceraldehyde-3-phosphate, a key reaction in the glycolysis pathway. TPI has been previously identified as the most abundantly secreted protein by *Brugia malayi*, the causative agent of human lympathic filariasis, and is required for optimal fecundity^74^. TPI is also highly secreted by other parasites, such as the nematodes *Meloidogyne incognita*^75^ and *Haemonchus contortus*^76^, and the trematode *Schistosoma mansoni*^77^. It remains unclear, however, how this enzyme alters the phenotype of host immune cells. Evidence for a moonlighting function of TPI has been reported for *Trichomonas vaginalis*^78^ and the fungal pathogen *Paracoccidioides brasiliensis*^79^, where surface-associated TPI interacts with extracellular matrix components such as laminin and fibronectin to facilitate adhesion and invasion. Although this alternate function for TPI does not address molecular mechanisms implicated in immunomodulation, it does suggest that uncharacterized and perhaps unexpected roles for such enzymes in the infection process.

The cytoplasmic enzyme NDK catalyzes the exchange of a terminal phosphate from a donor NTP to an acceptor NDP to produce a triphosphate, a necessary step for the synthesis of DNA and RNA. Interestingly, NDK also is released into the extracellular milieu by parasites and other microbes and contributes to the inhibition of innate immune responses^80^. It is possible that the *T. suis* NDK exerts its effect by degrading extracellular ATP (eATP) to reduce purinergic signaling through P2X_7_ receptors and inflammasome-mediated responses, preventing release of pro-inflammatory cytokines, as observed with other pathogens^80,81^. Moreover, eATP levels affect macrophage polarization; higher levels of this eATP favor M1 polarization^82,83^.

Despite their striking effects on innate immune cells, how these recombinant proteins or other proteins in TsESP exert immunomodulatory effects remains unclear. It is tempting to postulate that a mechanism involving the cross-linking of surface molecules/receptors that triggers a signaling cascade may lead to the internalization of *T. suis* proteins. For instance, C-type lectin receptors (CLR), pattern-recognition receptors (PRRs) expressed on DCs and macrophages, are involved in the recognition and internalization of carbohydrates and polypeptides^84^. The *Mycobacterium*-derived mannose-capped lipoarabinomannans (ManLAMs) bind to the mannose receptor DC-SIGN to trigger an inhibitory signal that blocks LPS-induced activation and suppress dendritic cell functions and IL-12 secretion^85,86^. Helminths express glycosylated molecules, several of which bind to innate receptors and modulate host immune responses^87^. *Schistosome* soluble egg antigens (SEA), a complex mixture of glycosylated proteins and lipids, are internalized by dendritic cells through different CLR, namely DC-SIGN, MGL, and the mannose receptor, and to co-localize with MHC-II-positive lysosomal compartments^88^. It will also be interesting to determine if similar mechanisms are implicated in the response to TsESP exposure, but also how these molecules block TLR ligand-mediated and cytokine-induced inflammatory responses.

Understanding the nature of the molecular dialogue between the parasite and its host that drives immune modulation could lead to the development of new strategies for combating autoimmune disorders. A focused and targeted approach in the treatment of these debilitating diseases using parasite-derived molecules will circumvent the disadvantages associated with the inoculation of patients with live organisms.

## Materials and Methods

### Isolation of *T. suis* life cycle stages

Pig (*Sus scrofa*) management and handling procedures were approved by the Beltsville Area Animal Care and Use Committee (Protocol #10-011) following Institutional Animal Care and Use Committees (IACUC) guidelines. Mixed-sex pigs (crossbred: Landrace × Yorkshire × Poland China) of approximately 3 months of age were inoculated with a single dose of infective *T. suis* eggs (2 × 10^4^ eggs/pig), as previously described^9^. Swine were housed in stalls with a nonabsorptive concrete floor surface, two pigs per pen, and had access to water and feed ad libitum. The diet was a corn-soybean formulation containing 16% crude protein and vitamins and minerals that exceeded National Research Council guidelines^9^. Following euthanasia, *T. suis* larval stages and adult worms were collected at various time points, between day 10 to 42 post-inoculation from the cecum and proximal colon. Larvae and adult worms were collected and pooled from 2 to 9 infected pigs (average of 4–5 pigs for each parasite stage, for each infection trials). Larval stages were isolated after pig tissue was washed free of adherent fecal material with warm tap water and then incubated for 3 h in PBS. Adult worms were manually removed from the tissue using fine forceps. 10- and 16-day larvae were freed of debris by floatation over a 40% Percoll; 21- and 28-day larvae were collected by swirling around a curved Pasteur pipette; 42-day adult worms (L5) were picked individually free of debris. Worms were washed five times, for 10 min each, by sedimentation in wash buffer (Hank’s Balanced Salt Solution (HBSS) pH 7.2 supplemented with 500 U/mL penicillin, 500 µg/mL streptomycin, 1.25 µg/mL amphotericin B, and 350 µg/mL chloramphenicol) (Gibco, Carlsbad, CA) at a ratio of 10 parts wash buffer to 1 part worms. Pigs were free of other helminth infections as measured by routine screening of the herd and the absence of worms in the intestinal contents and liver lesions at necropsy.

### Production of *T. suis* excretory-secretory protein (TsESP)

Parasites were incubated in wash buffer O/N at 37 °C, 10% CO_2_ in air then resuspended in culture medium (DMEM with 4.5 g/mL glucose, L-glutamine, and sodium bicarbonate at pH 7.2, 250 U/mL penicillin, 250 µg/mL streptomycin, 0.625 µg/mL amphotericin B, and 400 µg/mL chloramphenicol) (Sigma-Aldrich, St. Louis, MO) at a concentration of ~20 adult worms/mL or ~500 larvae/mL and incubated at 37 °C, 5% CO_2_. Culture medium was replaced every 24 h for a period up to 72 h, then worms were collected by filtration using a Whatman filter paper. Conditioned culture media were sterilized by filtration through a 0.22 µm Amicon bottle top filter (Millipore, Billerica, MA). TsESP were concentrated under nitrogen pressure (55 psi) using a stirred ultrafiltration chamber (Amicon model 8400, Millipore) with a Millipore Ultracel 3 kDa MWCO ultrafiltration disc. Protein concentrations were measured using the RCDC assay (Bio-Rad, Hercules, CA), according to the manufacturer’s specifications, and the samples were aliquoted and stored at −80 °C.

### Genome sequencing, assembly, and annotation

Genomic DNA (gDNA) from the different *T. suis* life stages (mixed sex) was extracted and purified using the kit E.Z.N.A. SQ Tissue DNA Kit (Omega Bio-tek, Norcross, GA), and the yield and purity assessed by Bioanalyzer (Agilent Technologies, Santa Clara, CA). Whole-genome shotgun fragment and paired-end sequencing libraries (3 kb and 8 kb) were constructed from the gDNA, as described^89^, and sequenced on the Illumina HiSeq. 2000 platform (392x). Linker and adapter sequences were trimmed, and cleaned reads were assembled using ALLPATHS-LG^90^. A repeat library was generated using Repeatmodeler (http://www.repeatmasker.org/RepeatModeler.html), rRNA genes were identified using RNAmmer (http://www.cbs.dtu.dk/cgi-bin/nph-sw_request?rnammer), and tRNAs were identified with tRNAscan-SE^91^. Non-coding RNAs were identified by sequence homology search of the Rfam database (http://selab.janelia.org/software.html). Repeats and predicted RNAs were then masked using RepeatMasker^92^. Repeat count data are available in Supplementary Table S6. A total of 9,832 protein coding genes as predicted using a combination of the *ab initio* programs Snap^93^, Fgenesh^94^, and Augustus^95^, and the MAKER annotation pipeline^96^. A consensus gene set based on these predictions was generated using a hierarchical approach developed at The Genome Institute^97^ and gene product naming was determined by BER (http://ber.sourceforge.net). Transmembrane domains and classical secretion peptides were predicted using Phobius^98,99^, and non-classical secretion signals were predicted using SecretomeP^100^. Proteins were assigned to KEGG orthologous groups, pathways and pathway modules using KEGGscan^101^ with KEGG release 68^102^. Associations with InterPro protein domains and Gene Ontology (GO) classifications were inferred using InterProScan^103–105^. All available functional annotations are available in Supplementary Table S3. Functional enrichment of GO terms related to particular subsets of proteins was calculated using FUNC^106^ with an adjusted *P*-value cutoff of 0.01 (Supplementary Table S4). Orthologous protein families were defined using the OrthoMCL package^107^ with an inflation factor 1.5, based on the complete deduced proteomes from the *T. suis* genome annotation, and of *T. muris*, *T. trichiura*^61^, and host species *Homo sapiens*, *Mus musculus*, and *Sus scrofa* (retrieved from Genbank, January 2014)^108^.

### Transcriptome sequencing

Paired-end cDNA libraries were generated as previously described using standard protocols^109^ from mRNA preserved from freshly isolated *T. suis* parasites from different life stages (2 replicates for each larval stage and 3 for adult worms). Adapter trimming, sequence quality trimming, length filtering, complexity filtering, and contaminant filtering were performed as previously described^110^. The remaining high-quality RNA-Seq reads were aligned to the genome assembly using Tophat2^111^ (version 2.0.8, default parameters). The mean number of reads associated with each feature for the replicates was determined using HTSeq-Count^112^ (Supplementary Tables S3, S4). Differentially expressed genes were predicted using DESeq2 (version 1.4.5)^113^ with an adjusted *P*-value cutoff of 0.05. All processed gene expression data and differential expression data is available in Supplementary Table S3.

### Liquid chromatography-coupled tandem-mass spectrometry (LC-MS/MS)

LC-MS/MS analysis was performed at the Proteomics Platform of the Quebec Genomics Centre (Quebec City, QC, Canada). Proteins were solubilized in 25 µL 50 mM NH_4_HCO_3_, 1% sodium deoxycholate, 0.2 mM DTT, and 0.9 mM iodoacetamide, and digested with sequencing-grade trypsin (Promega, Madison, WI) for 16 h at 37 °C. Peptides were concentrated using a Stage tip (C18), vacuum-dried, and resuspended in 5 µL 0.1% formic acid, then resolved by reverse-phase (RP) on self-packed PicoFrit column (New Objective, Woburn, MA) packed with Jupiter (5.0 u, 300 Å C_18_, 15 cm × 0.075 mm internal diameter) (Phenomenex, Torrance, CA) with a linear gradient from 2 to 50% Solvent B (ACN, 0.1% formic acid) over 90 min, at 300 nL/min. Full survey spectra were collected (400 to 2,000 m/z) and analyzed on a 5600 mass spectrometer using Analyst (version 1.6) (AB Sciex, Framingham, MA), and the seven most intense ions were submitted to fragmentation. Spectra were searched against a database of predicted tryptic peptides derived from the predicted protein sequences translated from the *T. suis* genome annotation using MASCOT version 2.3.02 (Matrix Science, London, UK). MS data was analyzed with Scaffold (version 4.0.1, Proteome Software Inc., Portland, OR) with a peptide confidence of 95.0% with a minimum of 1 peptide in each sample for a given life-stage.

### Murine bone marrow-derived immune cell cultures

BMDC were generated from 6–8 week-old female C57BL/6 mice. Mice were maintained in the animal facility at the Lyman-Duff Medical Building (McGill University, Montreal, QC Canada) according to the McGill University Animal Care Committee (Permit #4543). This protocol respects the procedures on good animal practice provided by the Canadian Council for animal care. BMDC were obtained by differentiating precursor cells as previously described^22^. Bone marrow cells (2.5 × 10^5^ cells/mL) were differentiated for 7 days in complete medium (RPMI 1640 supplemented with 5% heat-inactivated FBS, 10 mM HEPES, 2 mM L-glutamine 20 µg/mL gentamycin, and 50 µM β-ME) (Wisent, St-Bruno, QC, Canada) enriched with 20% Ag8.653-conditioned culture medium. CD11c^+^ BMDC were purified by positive selection using anti-CD11c MACS beads (Miltenyi Biotec, Auburn, CA). Purity was assessed routinely by flow cytometry, monitoring CD11c surface expression. BMDM were obtained by differentiating precursor murine bone marrow cells (5 × 10^6^) resuspended in culture medium (DMEM, 10% FBS, 2 mM L-glutamate, 100 U/mL penicillin, 100 µg/mL streptomycin, 50 µg/mL gentamicin, 2.5% HEPES, 55 µM β-ME, 1 mM sodium pyruvate) (Wisent) supplemented with 30% L929 fibroblast-conditioned culture medium for 7 days^114^. Purity was assessed routinely by flow cytometry, monitoring CD11b and F4/80 co-expression.

### Cell culture treatments and stimulation

BMDM and BMDC were plated at 2 × 10^5^ or 2 × 10^6^ cells/well in 96 or 6-well plates, respectively, and cultures were treated with TsESP or recombinant proteins or left untreated for 4 h. Fresh medium was added with or without CpG-ODN (1 µM final) (InvivoGen, San Diego, CA) to BMDC or recombinant murine IFNγ (BioSource, Carlsbad, CA) and LPS from *Salmonella enterica* (Sigma-Aldrich) (100 U/mL and 10 ng/mL final, respectively), IL-4 (Peprotech, Rocky Hill, NJ) and IL-13 (BioLegend, San Diego, CA) (both 10 ng/mL final), or with IL-4, IL-13, and IL-10 (eBioscience, San Diego, CA) (all 10 ng/mL final) to BMDM, and cultures were incubated for 18–20 h.

### ELISA

Secreted cytokines in culture supernatants from treated BMDC or BMDM cultures were measured by sandwich ELISA. Plates were coated with the following capture antibodies: anti- IL-10 (clone JES5–16E3), anti-IL-12p35 (clone C18.2), anti-TNFα (clone 1F3F3D4) (eBioscience), anti-IFNγ (clone R4–6A2), and anti-IL-4 (clone 11B11) (BioLegend). Wells were blocked with PBS 0.05% Tween 20 (PBS-T) with 1% BSA (Sigma-Aldrich) for 1 h at room temperature (RT), then culture supernatants were added to wells and incubated O/N at 4 °C. On the following day, the following biotinylated antibodies were used: anti-IL-10 (clone JES5-2A5), anti-IL-12/IL-23p40 (clone C17.8), anti-TNFα (clone XT3/XT22) (eBioscience), anti-IFNγ (clone XMG1.2), and anti-IL-4 (clone BVD6–24G2) (BioLegend). Concentrations were calculated from standard curves generated using linear regression analysis of data obtained from serial dilutions using recombinant murine IL-10, IL-12p70, TNFα (eBioscience), IL-4, and IFNγ (BioLegend) standards.

### Western blot analysis

Treated cells were lysed in ice-cold RIPA buffer (25 mM Tris (pH 7.6), 150 mM NaCl, 1% Triton-X 100, 0.5% sodium deoxycholate, 0.1% SDS) supplemented with phosphatase and EDTA-free protease inhibitor cocktails (Roche, Basel, Switzerland). Insoluble material was removed by centrifugation and protein concentration measured using the BCA assay (Pierce, Rockford, IL). Proteins were resolved by SDS-PAGE and then transferred to PVDF membranes (Bio-Rad). Membranes were blocked and probed with the primary antibodies: anti-NOS2 (eBioscience), anti-arginase-1 (Arg1) (Santa Cruz Biotechnology, Santa Cruz, CA), anti-phospho-STAT3 (Y705), anti-STAT3, anti-C/EBPβ, anti-TTP, anti-β-actin (Cell Signaling Technologies, Danvers, MA), or anti-NFIL-3 (BioLegend) and goat anti-rabbit or goat anti-mouse (Sigma-Aldrich) IgG horseradish peroxidase (HRP)-linked antibodies. Proteins were visualized using the Clarity ECL Western blotting substrate (Bio-Rad).

### Nitric oxide (NO) production and arginase enzymatic activity

Culture supernatants were collected and NO production was determined using the Griess reagent according to manufacturer’s specifications (Biotium, Hayward, CA), while cells were kept to test for arginase activity (see below). Absorbance was measured at 548 nm with a Synergy H4 (Biotek) plate reader, and nitrite concentrations were calculated using linear regression obtained from serial dilution of nitrite standards. Arginase activity was measured using the colorimetric reaction between α-ISPP (Sigma-Aldrich) and urea, a technique adapted from previously published work^115^. Color development was measured at 540 nm using a Synergy H4 (Biotek) plate reader, and arginase enzymatic activity was calculated using values obtained from urea standards and expressed as milli-enzyme units per million cells (mU/10^6^ cells).

### Flow cytometry staining

Following experiment-specific treatments, cells were harvested, washed, and resuspended in FACS buffer (PBS with 0.1% BSA). When required, cells were first stained with fixable viability dye (eFluor780; San Diego, CA). Fc receptors were blocked with anti-mouse CD16/32 (Fcγ III/II) (clone 2.4G2; BD Biosciences, San Jose, CA), then stained with the following antibodies, according to the experiment, on ice for 30 min: PerCP-eFluor710-anti-mouse CD4 (clone GK1.5), and PE-anti-mouse-CD11c (clone N418) (eBioscience). For intracellular cytokine staining, cells were fixed and permeabilized using FoxP3/Transcription Factor Staining Buffer Set (eBioscience), according to the manufacturer’s specifications, then stained with the following antibodies: FITC-anti-mouse-IFNγ (clone XMG1.2), APC-anti-mouse-TNFα (clone MP6-XT22), APC-anti-mouse-IL-10 (clone JES5.16E3) (eBioscience). UltraComp eBeads (eBioscience) were stained with each fluorochrome in the panel of markers as compensation controls. Samples were acquired using either a BD FACSCalibur or Fortessa (BD Biosciences), and data were analyzed using FlowJo software (Tree Star, Ashland, OR).

### *In vitro* suppression assay

Purified CD11c^+^ BMDC (5 × 10^4^/100 µL) were pretreated with *T. suis* ESP (50 µg/mL) for 2 h or medium alone. Splenic CD4^+^ cells from OT-II mice were negatively selected using a mouse CD4^+^ T cell isolation kit (StemCell Technologies, Vancouver, BC) and labeled with CFSE, as previously described^116^. CFSE-labeled CD4^+^ T cells (2.5 × 10^5^/100 µL) were added to CD11c^+^ BMDC cultures (i.e. T cell-to-BMDC ratio of 5:1) and stimulated with 1 nM OVA_323–339_ peptide (AnaSpec, Fremont, CA). After 72 h, cells were harvested and processed for flow cytometry analysis; CFSE dilution was determined in gated CD4^+^ T cells. Supernatants from the co-cultures were collected and assayed for IFNγ, TNFα, and IL-10 by sandwich ELISA using standard protocols and DuoSet ELISA antibodies (R&D Systems, Minneapolis, MN).

### Intracellular cytokine staining (ICS)

Co-cultures of BMDC and OT-II CD4^+^ T cells containing CD11c^+^ BMDC pretreated with larvae or adult worm *T. suis* ESP, or left untreated for 2 h and then 5 × 10^6^ purified OT-II CD4^+^ T cells were added in the presence of 1 nM OVA_323–339_ peptide (Anaspec). Cells were cultured at 37 °C, 5% CO_2_ for 48 h, and Brefeldin A (eBioscience) was added during the last 4 h of culture to inhibit cytokine secretion. Non-adherent T cells were harvested and adherent BMDC were collected using 1X TrypLE (Invitrogen); cells were processed separately for flow cytometry analysis.

### Gel permeation chromatography

Gel permeation chromatography was performed on a Beckman Coulter System Gold (Beckman Coulter, Brea, CA) equipped with a Superdex 200 gel permeation chromatography column (GE Healthcare Life Sciences, Marlborough, MA) equilibrated with endotoxin-free PBS (pH 7.4–7.5). Approximately ~4.0 mg of adult worm TsESP was injected onto the column and fractionated at a flow rate of 0.5 mL/min, and 1 mL fractions were collected. Eluting proteins were monitored at 280 nm wavelength. The column was calibrated using a standard protein mixture containing thyroglobulin (670 kDa), bovine IgG (158 kDa), ovalbumin (44 kDa), equine myoglobin (17 kDa), and vitamin B12 (1.35 kDa) (Bio-Rad).

### Recombinant protein expression

Genes encoding the recombinant *T. suis* triosephosphate isomerase (TPI, D918-00560), nucleoside diphosphate kinase (NDK, D918-00383), and small nuclear ribonucleoprotein (SNRP, D918-00505) 505 were obtained as a synthetic gene from GenScript cloned into expression vector pET15b vector (Millipore). *E. coli* ER2566 (New England Biolabs) transformed with the pET15b-*TPI*, pET15b-*NDK*, or pET15b-505 vectors were grown at 37 °C and protein expression was induced with 15 mM benzyl alcohol and 0.2 mM IPTG. Bacterial cell pellets were resuspended in PBS (pH 7.2) and cells were lysed by sonication, NaCl was added (0.5 M final) and clarified supernatants were applied to Ni-NTA columns. Columns were washed with PBS, followed by 50 mL of PBS with 2% Triton X-114 to remove endotoxins, then with 25 mL of PBS. Recombinant proteins were eluted with a 80, 160, and 320 mM imidazole step gradient in PBS. Fractions containing a homogeneous preparation were concentrated using an Amicon ultracentrifugal filter unit, and the buffer was exchanged for PBS.

### Statistical analyses

Where applicable, statistical significance was determined using one-way ANOVA followed by Bonferroni post-hoc test; calculations were performed using Prism software (GraphPad Software, La Jolla CA). Differences were considered significant when *P < 0.05, **P < 0.01, ***P < 0.001, while not significant differences are indicated by “*ns*”.

## Electronic supplementary material

Table S5

Table S4

Table S3

Table S2

Supplementary Information

## Data Availability

Raw reads from transcriptomic analyses were deposited in the GenBank Sequence Read Archive under BioProject ID PRJNA179528 (SRX1838974 - SRX1838986). Genomic sequencing and gene annotation data are available on the NCBI sequence read archive (Biosample ID SAMN02721381, BioProject ID PRJNA179528). All other datasets generated and analysed during the current study are available upon request.
