# Supplementary material for: Analysis of the Trichuris suis excretory/secretory proteins as a function of life cycle stage and their immunomodulatory properties
Source: Sci Rep. 2018 Oct 29;8:15921. doi: 10.1038/s41598-018-34174-4 (PMC6206011; doi:10.1038/s41598-018-34174-4)
Supplement: Supplementary file 5 — Supplementary Information [file 41598_2018_34174_MOESM5_ESM.docx]

**SUPPLEMENTARY INFORMATION**

**Analysis of the *Trichuris suis* excretory/secretory proteins as a function of life cycle stage and their immunomodulatory properties**

Louis-Philippe Leroux, Mohamad Nasr, Rajesh Valanparambil, Mifong Tam, Bruce A. Rosa, Elizabeth Siciliani, Dolores E. Hill, Dante S. Zarlenga, Maritza Jaramillo, Joel V. Weinstock, Timothy G. Geary, Mary M. Stevenson, Joseph F. Urban Jr., Makedonka Mitreva, Armando Jardim

**Supplementary Materials and Methods**

**Proteolytic degradation of TsESP proteins**

TsESP were mixed with 5 µg/mL proteinase K (Roche) and incubated at 56°C for 16 h. The reaction was stopped by boiling samples for 15 min. Proteinase K without any protein were included as a control.

**Splenocyte differentiation into T_reg_**

For induction of T regulatory (T_reg_) cells by TsESP treatment *ex vivo*, splenocytes from C57BL/6 mice were resuspended in culture medium (RPMI, 10% FBS, 100 U/mL penicillin, 100 µg/mL streptomycin, 55 µM β-ME, 1 mM sodium pyruvate (Wisent) and plated at 2 × 10^6^ cells/well in 24-well plates. Cells were allowed to settle for 1 h at 37°C, 5% CO_2_, then treated with larvae or adult TsESP (50 µg/mL), *H. polygyrus* ESP [^23^](#_ENREF_23) (50 µg/mL), or left untreated for 2 h. Cultures were treated with 2 µg/mL concanavilin A and collected after 48 h. Expression of CD4, CD25, and FoxP3 were assessed by flow cytometry using the following antibodies: FITC-anti-mouse CD4 (clone GK1.5), PE-anti-mouse CD25 (clone RM4-4) (eBioscience), and APC-anti-mouse FoxP3 (clone FJK-16s) (eBioscience).

**Supplementary Figures and Legends**

**
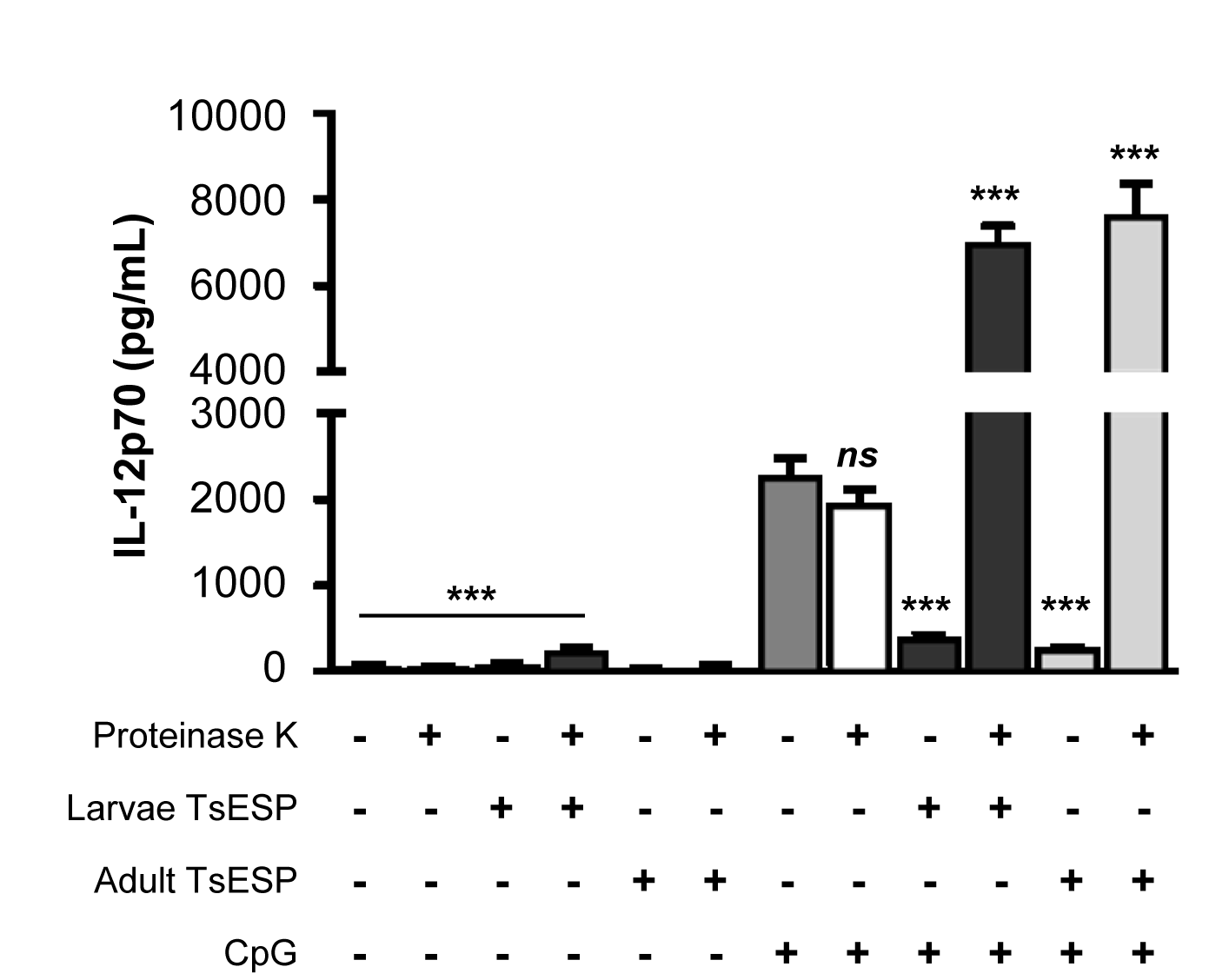
**

**Supplementary** **Figure S1: Proteolysis of TsESP abrogates immunosuppressive affect.** TsESP from 28-day larvae or adult worms were treated with 5 µg/mL proteinase K O/N, at 56°C. The reaction was stopped by boiling samples (~95-100°C) for 15 min. BMDC cultures were treated with either native or proteinase K-treated TsESP for 4 h, then stimulated with 1 µM CpG-ODN or left unstimulated for 18-20 h. Cultures were treated with proteinase K only as a control. Culture supernatants were collected and IL-12p70 concentrations were determined by ELISA. All samples were done in triplicates, and error bars represent standard deviation (SD). Statistical significance was calculated using one-way ANOVA (where *ns* = not significant, * *P* < 0.05, and ** *P* < 0.01).

**
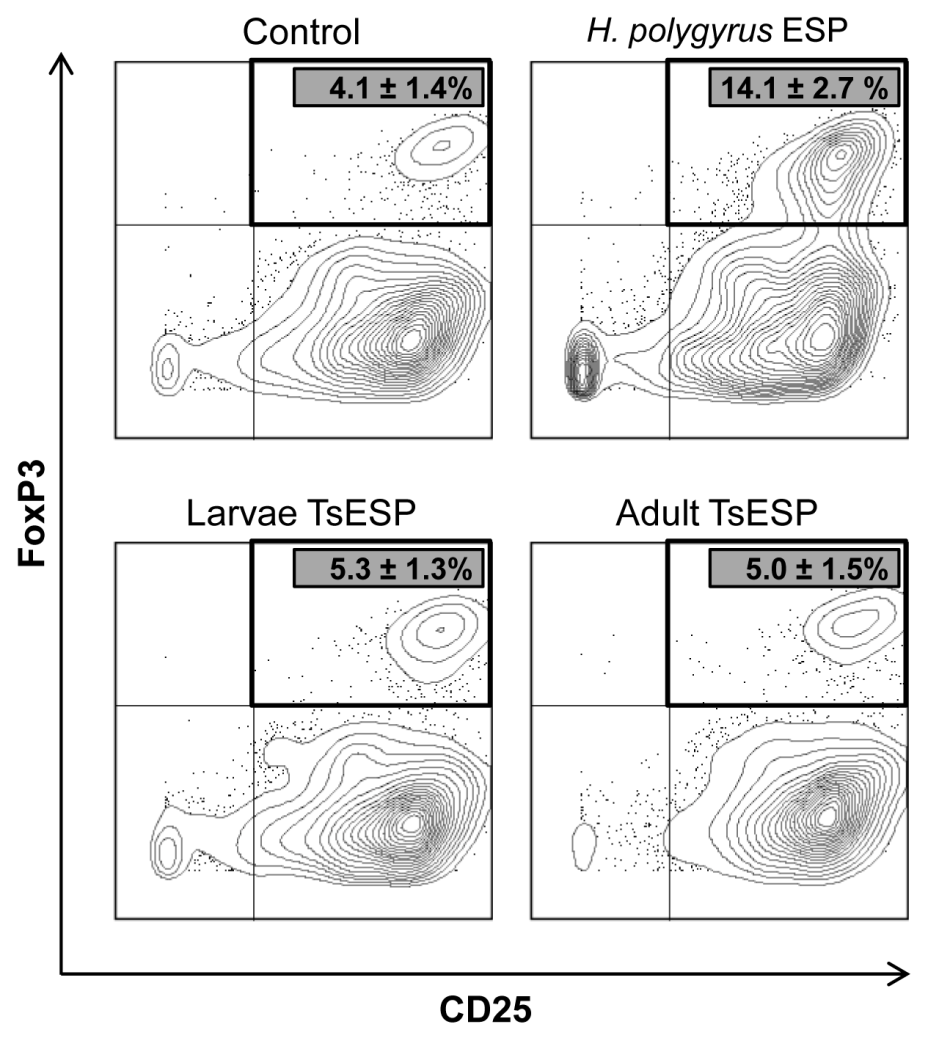
**

**Supplementary Figure S2: TsESP do not directly induce CD4^+^ CD25^+^ FoxP3^+^ T regulatory (T_reg_) cells.** Naïve splenocytes from C57BL/6 mice were cultured, treated with 50 µg/mL larvae or adult TsESP, 50 µg/mL *H. polygyrus* ESP, or left untreated for 2 h. Cells were then stimulated with 2 µg/mL concanavalin A (Con A) for 48 h. Phenotypic markers (CD4, CD25, and FoxP3) were assessed by flow cytometry. Gating was first performed to identify CD4^+^ cells, and CD25 and FoxP3 expression are displayed for the latter population. Contour levels on graphs are shown at 5% intervals. Values displayed here are from one representative experiment from two independent trials.


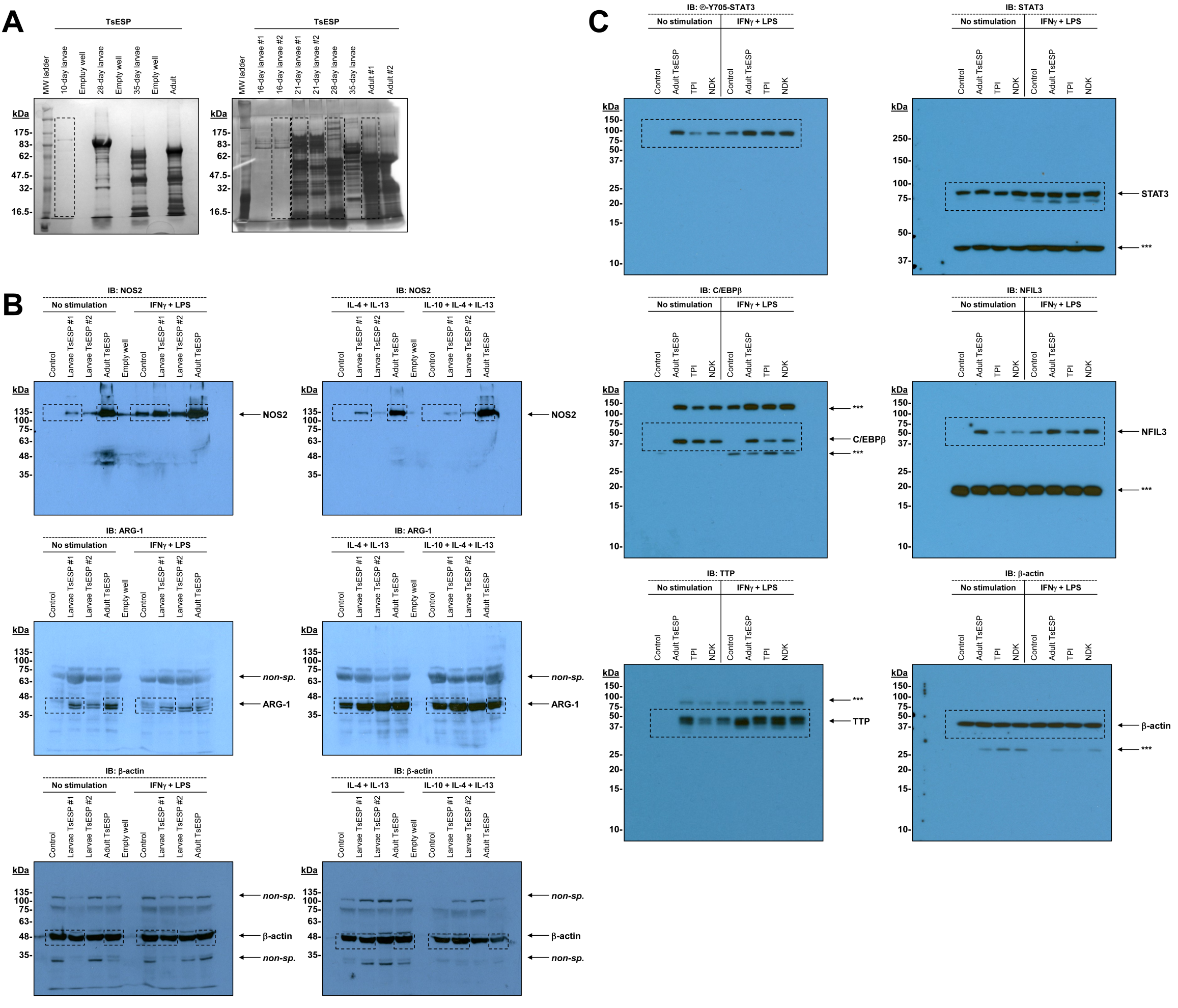


**
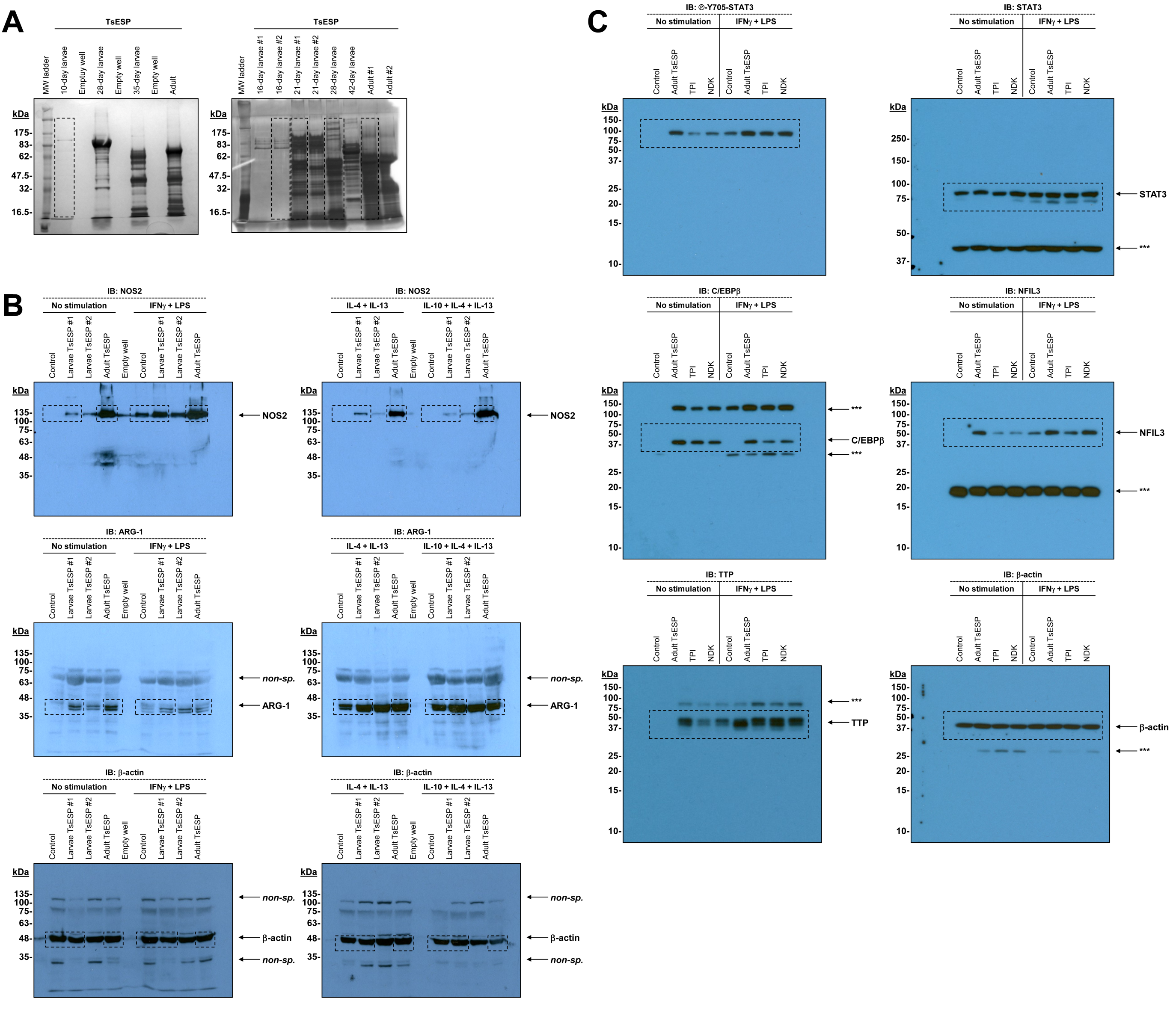
**

**Supplementary** **Figure S3: Full-length SDS-PAGE gel and Western blotting film scans.** Full-length scans for **(A)** silver-stained SDS-PAGE gels and **(B-C)** Western blotting films used to generate **Fig. 1A**, and **Fig. 4A** and **Fig. 7B**, accordingly. Dashed-line boxes indicate cropped areas used for the main figures. The labeling "#1" and "#2" correspond to sample batch numbers (i.e. biological replicate). Molecular weights (MW) in kDa are indicated on the side of the scans. **(B-C)** Antibodies used to probe the membranes are indicated for each panel (immunoblotting, IB), and specific signals corresponding to the proteins of interest are indicated by an arrow. **(B)** Non-specific signals due to antibody cross-reactivity are indicated by an arrow with the label "*non-sp.*". **(C)** Signals originating from previous probings with antibodies against non-related proteins (not non-specific signals) are indicated by an arrow with the label "***".

**Supplementary Tables**

**Supplementary** **Table S1: *T. suis* genome statistics and comparison with other *Trichuris* spp and with previous *T. suis* genomes*.***

| **Organism** | | Current study *T. suis* | | Previous *T. suis* genomes | | | Other *Trichuris* species | |
| --- | --- | --- | --- | --- | --- | --- | --- | --- |
|  |  |  |  | Female | | Male | *T. muris* | *T. trichiura* |
| **Contig Data** | total number | | 324 | | 3284 | 4292 | 1683 | 4156 |
|  | total length (bp) | | 63,932,509 | | 71,056,402 | 74,234,559 | 84,674,602 | 75,496,503 |
|  | avg length (bp) | | 197,323 | | 21,637 | 17,296 | 50,312 | 18,166 |
|  | median length (bp) | | 4,382 | | 330 | 314 | 1,914 | 3,965 |
|  | Max length (bp) | | 4,309,324 | | 1,448,326 | 1,594,463 | 1,774,400 | 533,758 |
|  | Min length (bp) | | 903 | | 201 | 201 | 511 | 101 |
|  | N50 length (bp) | | 1,322,386 | | 443,734 | 503,034 | 400,602 | 70,602 |
|  | N50 number | | 16 | | 46 | 44 | 59 | 265 |
|  | N90 length (bp) | | 243,854 | | 104,007 | 81,029 | 80,325 | 8,602 |
|  | N90 number | | 60 | | 168 | 185 | 229 | 1,435 |
|  | GC content | | 43.30% | | 43.50% | 43.60% | 44.80% | 42.20% |
|  |  | |  | |  |  |  |  |
| **Gene Data** | total number | | 9,832 | | 14,261 | 14,436 | 9,403 | 9,856 |
|  | avg exon size | | 196 | | 266 | 258 | 224 | 230 |
|  | avg intron size | | 215 | | 508 | 510 | 358 | 285 |
|  | avg gene length | | 2,384 | | 3,889 | 3,811 | 3,126 | 2,639 |
|  |  | |  | |  |  |  |  |
| **BUSCO** | Complete | | 284 | | 282 | 285 | 281 | 273 |
| **(genome** | Fragmented | | 4 | | 5 | 2 | 5 | 10 |
| **completeness)** | Missing | | 15 | | 16 | 16 | 17 | 20 |
|  | % | | 95.0% | | 94.7% | 94.7% | 94.4% | 93.4% |

**Supplementary** **Table S2: LC-MS/MS analysis of native TsESP from larvae and adult worms and functional annotation.** (Excel spreadsheet)

**Supplementary** **Table S3: Functional annotations, gene expression levels, and proteomic identifications for all *T. suis* genes.** (Excel spreadsheet)

**Supplementary** **Table S4: Gene Ontology (GO) enrichment (using FUNC) among sets of significantly differentially expressed genes.** (Excel spreadsheet)

**Supplementary** **Table S5: Proteins in gel permeation chromatography found in fractions displaying immunomodulatory activity.** (Excel spreadsheet)

**Supplementary** **Table S6: *T. suis* genome repeat sequence data.**

| **% of genome identified as repeat** |  |
| --- | --- |
| Total Masking (RM + trf) | 19.08% |
| RepeatMasker masking only | 18.31% |
| Tandem Repeat Finder (trf) only | 0.77% |
| **Classification of repeat library** |  |
| SINE | 0.12% |
| LINE | 1.89% |
| LTR elements | 0.94% |
| DNA elements | 4.52% |
| Unclassified | 10.75% |
| Small RNA | 0% |
| Simple Repeats | 0.08% |
| Low complexity | 0.11% |
